# Overexpression of Wnt5a promoted the protective effect of mesenchymal stem cells on Lipopolysaccharide-induced endothelial cell injury via activating PI3K/AKT signaling pathway

**DOI:** 10.1186/s12879-024-09204-4

**Published:** 2024-03-20

**Authors:** Manliang Guo, Shiqi Li, Chuan Li, Xueyan Mao, Liru Tian, Xintong Yang, Caixia Xu, Mian Zeng

**Affiliations:** 1 Department of Medical Intensive Care Unit, The First Affiliated Hospital, Sun Yat-Sen University, No. 58 Zhongshan Road 2, Guangzhou, Guangdong 510080 People’s Republic of China; 2 Research Center of Translational Medicine, The First Affiliated Hospital, Sun Yat-Sen University, No. 58 Zhongshan Road 2, Guangzhou, Guangdong 510080 People’s Republic of China; 3 Department of Urology, The Second Affiliated Hospital of Guangzhou Medical University, Guangzhou, Guangdong 510260 China

**Keywords:** Wnt5a, Mesenchymal stem cells, Endothelial cell injury, Acute lung injury, PI3K/AKT signaling

## Abstract

**Background:**

Lung endothelial barrier injury plays an important role in the pathophysiology of acute lung injury/acute respiratory distress syndrome (ALI/ARDS). Mesenchymal stem cells (MSCs) therapy has shown promise in ARDS treatment and restoration of the impaired barrier function. It has been reported that Wnt5a shows protective effects on endothelial cells. Therefore, the study aimed to investigate whether overexpression of Wnt5a could promote the protective effects of MSCs on Lipopolysaccharide (LPS)-induced endothelial cell injury.

**Methods:**

To evaluate the protective effects of MSCs overexpressing Wnt5a, we assessed the migration, proliferation, apoptosis, and angiogenic ability of endothelial cells. We assessed the transcription of protective cellular factors using qPCR and determined the molecular mechanism using Western blot analysis.

**Results:**

Overexpression of Wnt5a upregulated the transcription of protective cellular factors in MSCs. Co-culture of MSC^Wnt5a^ promoted endothelial migration, proliferation and angiogenesis, and inhibited endothelial cell apoptosis through the PI3K/AKT pathway.

**Conclusions:**

Overexpression of Wnt5a promoted the therapeutic effect of MSCs on endothelial cell injury through the PI3K/AKT signaling. Our study provides a novel approach for utilizing genetically modified MSCs in the transplantation therapy for ARDS.

**Supplementary Information:**

The online version contains supplementary material available at 10.1186/s12879-024-09204-4.

## Introduction

ALI/ARDS is a critical syndrome with diffuse pulmonary inflammation and exudation, leading to acute hypoxemic respiratory failure [1]. The prevalence of ARDS among intensive care unit admissions in 2016 was 10.4%, with hospital mortality ranging from 34.9% to 46.1% [2]. Thus, developing new therapies is imperative to improve the prognosis of ARDS. The classic pathological change in ARDS is the alveolar epithelial-endothelial barrier injury [3]. Sepsis, which results in diffuse damage to the vascular endothelium due to systemic inflammation, occurs in almost 80% of patients with ARDS [4, 5]. Disruption of endothelial junctions or endothelial cell death increases permeability to fluid and protein across the lung endothelium, resulting in edema in the lung interstitium [6]. Thus, endothelial cell injury is an important link in the pathogenesis of ARDS.

MSCs are extracted from bone marrow, fat and umbilical cord, and are widely utilized in cell therapy for inflammatory autoimmune diseases and tissue damage repair [7]. Increasing studies suggest that MSCs has significant therapeutic potential for ALI/ARDS by modulating crucial pathobiological pathways, such as anti-inflammation, alveolar-capillary barrier protection, surfactant production and apoptosis [8]. Importantly, MSC therapy has been confirmed effective for ARDS, including in severe COVID-19 patients [9–13]. To overcome the defects of MSC therapy, strategies such as genetic modification and precondition have been used to optimize MSC therapy for ARDS [14]. Our previous study demonstrated that pretreatment with ghrelin enhances the therapeutic value of MSCs on LPS-induced endothelial cell injury, and this is partially attributed to upregulation of Homeobox B4 (HOXB4) [15, 16]. Other than HOXB4, the transcriptome sequencing results also revealed a significant upregulation of Wnt5a in MSCs pretreated with ghrelin [15].

Wnt5a, a secreted glycoprotein, is a member of the noncanonical Wnt family [17]. Wnt5a is abundantly expressed in mouse limb mesenchyme and growth plate cartilage, and participates in MSCs differentiation [18, 19]. Upregulating Wnt5a expression will induce the osteogenic and chondrogenic differentiation of MSCs via different mechanisms [20, 21]. Additionally, previous studies have shown that Wnt5a plays a key role in vascular endothelium, Wnt5a deficiency was found to be associated with endothelial and vascular dysfunctions [22–25]. However, there is limited research on the role of Wnt5a in MSC therapy for ALI/ARDS. It remains unclear whether the protective effect of MSCs is partially mediated by Wnt5a, and the underlying molecular mechanism warrants further investigation. Here, we discovered that overexpression of Wnt5a promoted the therapeutic effect of MSCs on LPS-induced endothelial cell injury through activation of PI3K/AKT signaling.

## Materials and methods

### Cell culture

**Fig. 1:**
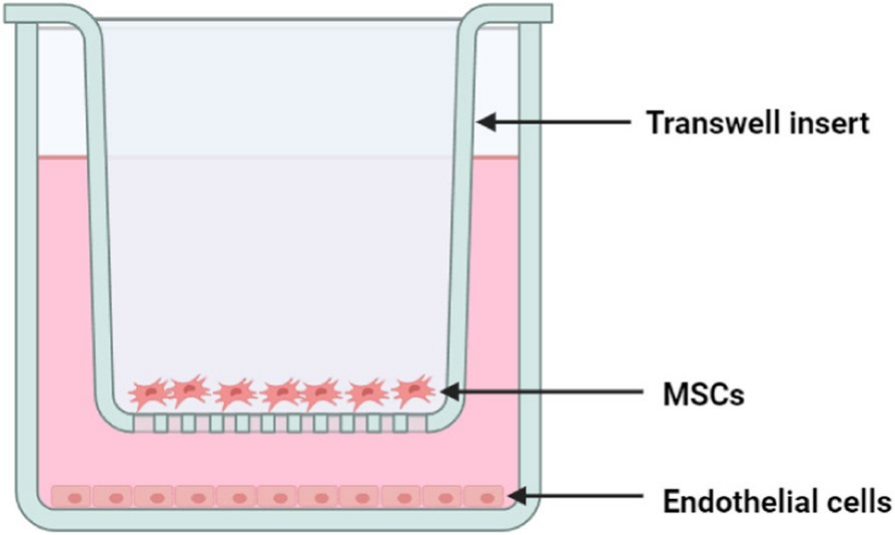
The co-culture system was established to investigate protective effects of MSCs on endothelial cells

C3H10T1/2 (ATCC, Manassas, VA) is a type of mouse embryonic MSCs and were cultured in Minimum Essential Medium Alpha (MEM-α), EA. hy926 endothelial cells were cultured in high glucose Dulbecco's modified Eagle's medium (DMEM). 10% fetal bovine serum (FBS) and 1% penicillin–streptomycin were added to the medium. MSC^Vector^ (C3H10T1/2 cells transfected with empty vector), MSC^Wnt5a^ (C3H10T1/2 cells transfected with Wnt5a gene) and endothelial cells were cultivated under optimal conditions at 37 °C with 5% CO_2_. Within a specialized co-culture system, MSC^Vector^ (1 × 10^5^ cells/mL) and MSC^Wnt5a^ (1 × 10^5^ cells/mL) were cultivated in the designated chamber, while endothelial cells (1 × 10^5^ cells/mL) were cultured in separate 6-well plates (Fig. 1). In accordance with our previous study, we stimulated endothelial cells with 150 μg/mL LPS (O127:B8, Sigma, USA) for 24 hours [26]. The study was divided into four groups: control, LPS (150 μg/mL), LPS + MSC^Vector^ (150 μg/mL), and LPS + MSC^Wnt5a^ (150 μg/mL). We used the classical PI3K/AKT inhibitor, LY294002 (Absin, China), to verify the underlying mechanism. LY294002 was applied at a concentration of 10 μM, as reported in a previous study [15].

### Flow cytometric assay

Flow cytometry was conducted to detect surface molecule expression of MSCs. MSCs were digested with Trypsin–EDTA solution and then centrifuged at 1100 rpm for 4 min. The cells were then washed twice and resuspended with pre-cooled PBS containing 1% bovine serum albumin (PBA). Next, MSCs were incubated with the following surface marker antibodies: CD29, CD34, CD45, and Sca1 (all from Biolegend, USA) in PBA at 4 °C for 30 min without light. After incubation, the uncombined antibodies were washed out and MSCs were analyzed using a CytoFLEX Flow Cytometer (BECHMAN COULTER, USA). The data were subsequently analyzed with FlowJo software (TreeStar Inc., Ashland, OR, USA).

### Multilineage differentiation assay

Osteogenic and adipogenic differentiation medium were used to assess the differentiation potential of MSCs. MSCs were cultured in osteogenic differentiation medium (QiDa, Shanghai, China) for two weeks to induce osteogenic differentiation, the medium was changed every other day, and the cells were stained with Alizarin Red S (Solarbio, China) to detect the formation of calcium nodules. To induce adipogenic differentiation, MSCs were grown in adipogenic differentiation medium (QiDa, Shanghai, China) for 2–3 weeks, and Oil Red O (Solarbio, China) was used to detect lipids in differentiated adipocytes.

### Lentiviral production and transfection of MSCs

MSCs were transfected with lentiviral particles containing the Wnt5a gene and empty vectors. The lentiviral system consisted of three vectors: the packaging plasmids psPAX2 (Addgene, USA), the envelope plasmids pMD2.G (Addgene, USA), and the expression vector pEGIP (Addgene, USA). To generate the Wnt5a expression vector, the cDNA encoding mouse Wnt5a (NM_009524.4) was amplified by RT-PCR using specific primers, digested with restriction enzymes, and subcloned into the expression vector plasmid pEGIP. The recombinant lentivirus was produced by co-transfecting psPAX2, pMD2.G, and the expression vector containing Wnt5a or empty vector into HEK 293 T cells using calcium phosphate transfection. The supernatant containing the virus was collected 48 h after transfection, centrifuged at 1,500 rpm for 5 min to remove debris, and then filtered through a 0.45 μm pore-size filter. For lentiviral transduction, MSCs were incubated with lentiviral solution and 10 μg/mL polybrene (Sigma-Aldrich, USA, H9268) in complete medium for 9 h. Subsequently, the culture medium was changed, and MSCs were selected in complete medium containing 1.6 μg/mL of puromycin (Genechem, China) for 72 h. Finally, the MSC^Wnt5a^ and MSC^Vector^ cell lines were obtained and expanded for subsequent experiments.

### Scratch wound healing assay

1 × 10^5^ endothelial cells were seeded in each well of 6-well plates and cultured until they reached confluence. Scratches were created along the ruler using sterile pipette tips (200 μL), and the wells were washed with PBS for three times to remove the detached cells. Subsequently, 1 mL DMEM without FBS was supplied and endothelial cells were cultured for 24 h in different systems based on the grouping. The scratches were photographed at 0 and 24 h, and the scratch areas were quantified using ImageJ software to evaluate the migratory capacity of endothelial cells in various groups. Wound healing percentage (%) = [(0 h scratch area) – (24 h scratch area)] / (0 h scratch area) × 100%. (0 h scratch area: the scratch area when the scratch was created; 24 h scratch area: the scratch area when endothelial cells were cultured for 24 h after the scratch was created).

### Cell proliferation assay

The Cell-Light EdUApollo567 In Vitro Kit (Ribobio, China) was utilized to assess the cell proliferation capacity of endothelial cells. 2 × 10^4^ endothelial cells were seeded in 24-well plates and cultured under various conditions based on the experimental groups. Following a 24h incubation, the culture medium was aspirated, and the endothelial cells were washed twice with PBS. The endothelial cells were treated with EDU medium for 2 h, followed by fixation in 4% paraformaldehyde for 30 min and incubation in a 2 mg/ml Glycine solution for 5 min to neutralize any residual aldehyde groups. Next, endothelial cells were incubated with 0.5% Triton X-100 to enhance the permeability of the cell membrane, and then treated with Apollo® staining reaction solution for 30 min in the absence of light. Next, the staining solution was removed by washing the cells three times with 0.5% Triton X-100. Subsequently, the cells were incubated with Hoechst 33,342 reaction solution for 30 min in the absence of light and washed away with PBS. All procedures were conducted at room temperature. Finally, the cells were maintained in a moist environment with PBS, and the fluorescence pictures of the endothelial cells were taken using a fluorescent microscope. The number of EdU-positive cells and DAPI-positive cells was quantified using ImageJ software. The cell proliferation ability was determined by calculating the percentage of EdU-positive cells.

### Apoptosis assay

Endothelial cell apoptosis levels were assessed by employing the Annexin V-FITC/PI Apoptosis Kit (Elabscience, China). The culture medium and endothelial cells were collected and centrifuged at 300 × g for 5 min. The cells were then washed twice with PBS and resuspended in 500 μl 1 × Annexin V Binding Buffer at a concentration of 2 × 10^6^ cells/ml. Afterwards, 2.5 μl of Annexin V-FITC Reagent and 2.5 μl of PI Reagent were added. The cells were then incubated in the dark at room temperature for 20 min. Finally, the proportions of Annexin V and PI-positive cells were analyzed using flow cytometry within 1 h.

### Tube formation assay

200 μl Matrigel (Corning, USA) was plated in the wells of 24-well plate, then the plate was placed at 4 °C overnight to make the Matrigel evenly distributed. 2 × 10^4^ endothelial cells was seeded on the Matrigel. Following a 4-h culture, the medium was replaced with fresh high-glucose DMEM to eliminate any unattached cellular debris. Subsequently, 10 μl of Calcein AM fluorescent dye (KeyGen, China) was added 10 min before fluorescence microscopy observation to improve the visibility of cell frame on Matrigel. Subsequently, images were captured using a fluorescence microscope. The angiogenesis ability of endothelial cells was quantified by counting branching points in the randomly selected fields from each group using ImageJ software.

### Real-time RT-PCR

Total RNA was extracted from MSCs using Trizol reagent (Invitrogen, USA) following the manufacturer's protocol. The concentration of the RNA was assessed by the A260/A280 ratio, the ratio between 1.8 and 2.0 was considered acceptable. Subsequently, 1 μg of RNA was reverse transcribed into cDNA using the cDNA Synthesis SuperMix (NovoProtein, China). Quantitative Real-time PCR (qPCR) was conducted on an ABI PRISM 7500 Fast Detection System (Applied Biosystems, Carlsbad, CA, USA) using the SYBR qPCR Mix (NovoProtein, China) following the standard protocol. Each sample was analyzed in triplicate for the expression of GAPDH, Wnt5a, VEGF, Ang-1, FGF-10, HGF and TGF-β1. The PCR primer sequences are listed below (Table 1):

**Table 1:** Quantitative PCR primers sequences

Genes	Sequence
GAPDH	Fwd:5′-ACTCTTCCACCTTCGATGC-3′
	Rev:5′-CCGTATTCATTGTCATACCAGG-3′
Wnt5a	Fwd:5′-CAAGGGCTCCTATGAGAGC-3′
	Rev:5′-GCCAGGTTGTATACTGTCCT-3′
VEGF	Fwd:5′-CTGCTGTAACGATGAAGCCCTG-3′
	Rev:5′-GCTGTAGGAAGCTCATCTCTCC-3′
Ang-1	Fwd:5′-AACCGAGCCTACTCACAGTACG-3′
	Rev:5′-GCATCCTTCGTGCTGAAATCGG-3′
FGF-10	Fwd:5′-ATCACCTCCAAGGAGATGTCCG-3′
	Rev:5′-CGGCAACAACTCCGATTTCCAC-3′
HGF	Fwd:5′-GTCCTGAAGGCTCAGACTTGGT-3′
	Rev:5′-CCAGCCGTAAATACTGCAAGTGG-3′
TGF-β1	Fwd:5′-TGATACGCCTGAGTGGCTGTCT-3′
	Rev:5′-CACAAGAGCAGTGAGCGCTGAA-3′

Abbreviations: *GAPDH* glyceraldehyde-3-phosphate dehydrogenase, *Wnt5a* Wingless-related integration site family member 5a, *VEGF* vascular endothelial-derived growth factor, *Ang-1* angiotensin-1, *FGF-10* fibroblast growth factor-10, *HGF* hepatocyte growth factor, *TGF-β1* transforming growth factor-beta1

### Western blot assay

Endothelial cells were lysed with RIPA buffer (Beyotime, China), protease and phosphatase inhibitors were also added. The lysates were then centrifuged at 12,000 rpm and 4 °C for 10 min. The protein supernatant was collected and quantified using a BCA Protein Assay Kit (Beyotime, China). The protein extracts were separated by SDS-PAGE and transferred to PVDF membranes. The proteins were then blocked with 5% skimmed milk for 2 h at 25 °C. Subsequently, the proteins were reacted with the following primary antibodies for 18 h at 4 °C: anti-GAPDH (1:1000, Cat# 5174S, Cell Signaling Technology), anti-AKT (1:1000, Cat# 4691S, Cell Signaling Technology, Danvers, MA USA), anti-phospho-AKT (1:1000, Cat# 4060S, Cell Signaling Technology), anti-BAX (1:1000, Cat# 5023S, Cell Signaling Technology), and anti-Bcl-2 (1:1000, Cat# 4223S, Cell Signaling Technology). After incubation with horseradish peroxidase-conjugated secondary antibodies (1:3000, Cat# 7074S, Cell Signaling Technology) for 1 h at room temperature, the bands were visualized using the Image Quant LAS 4000 system.

### Statistical analysis

Statistical analyses were conducted using GraphPad Prism8 software (GraphPad Software Inc, USA). All the results were expressed as mean ± SD. Student's t-test was conducted for the two experimental groups, while one-way ANOVA and Bartlett's test (corrected) were used for multiple comparisons. *P* < 0.05 was deemed statistically significant.

## Results

### Characterization of MSCs

**Fig. 2:**
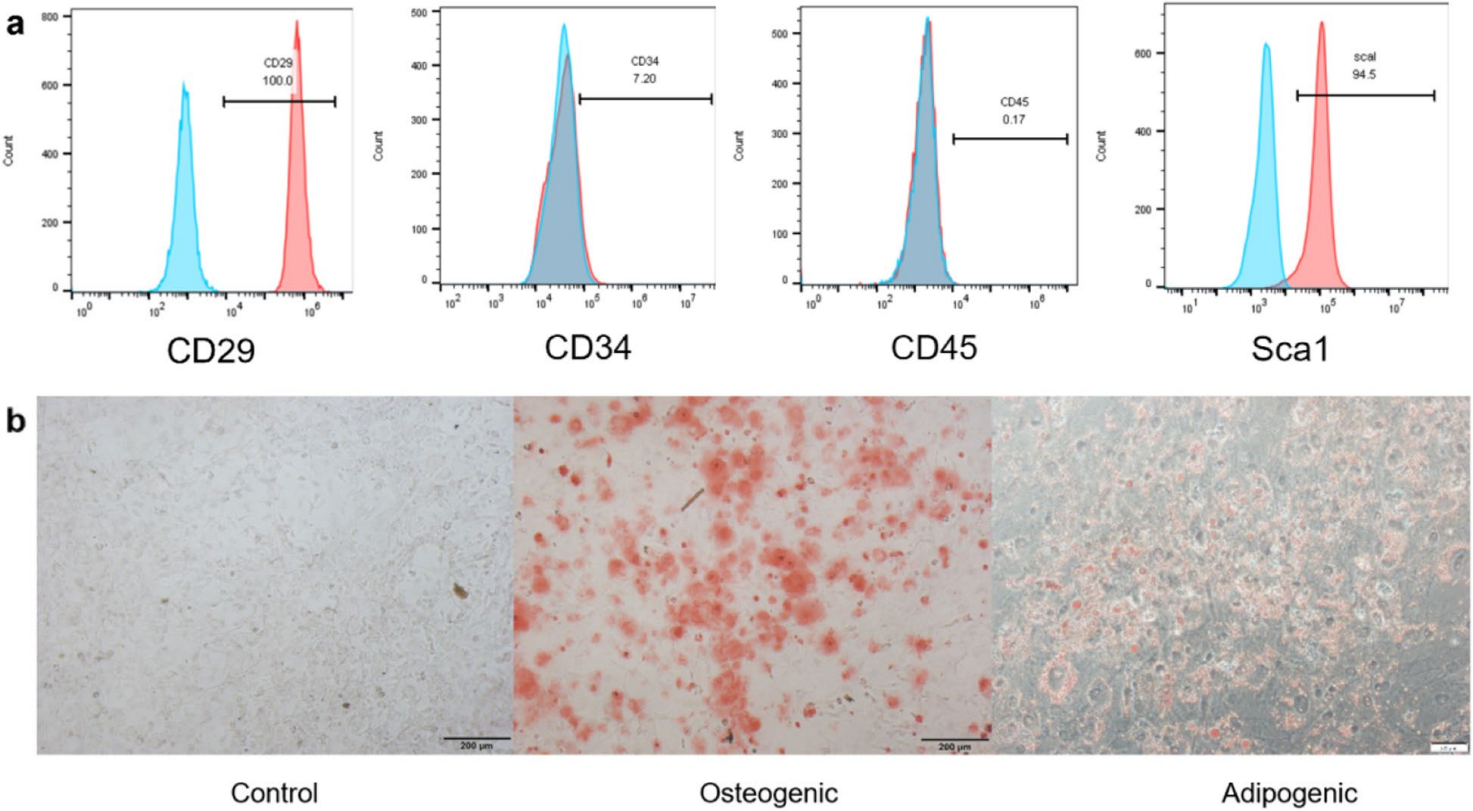
Characterization of MSCs. **a** MSCs were positive for CD29 and Sca1, but negative for CD34 and CD45. Unlabeled cells were used for negative control. The blue histogram represented the negative control, and the red histogram represented the expression of the surface marker. **b** Osteogenic differentiation capacity of MSCs in Osteogenic differentiation medium (middle) and adipogenic differentiation capacity of MSCs in adipogenic differentiation medium (right). Three independent replicates were performed

Flow cytometry was employed to identify the surface markers of MSCs, and the results were analyzed with FlowJo software. The findings indicated that MSCs expressed CD29 and Sca1, which are markers of stem cells, while they did not express CD34 and CD45, which are markers associated with endothelial cells or hematopoietic cells (Fig. 2a). Additionally, MSCs were cultured in conditioned medium to assess their multilineage differentiation potential in vitro. The findings showed that Osteogenic and adipogenic differentiation of MSCs could be detected via Alizarin Red S and Oil Red O (Fig. 2b).

### Successful overexpression of Wnt5a in MSCs

**Fig. 3:**
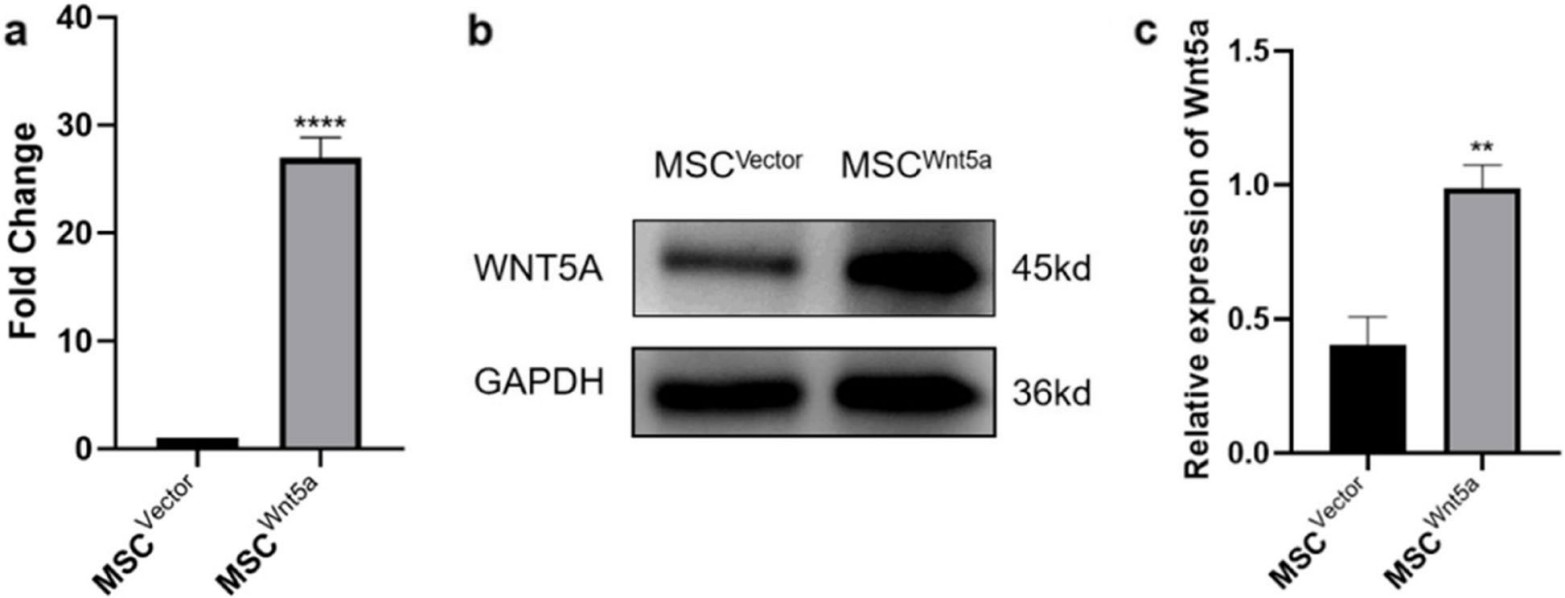
Validation of MSC^Wnt5a^. **a** Determination of Wnt5a mRNA transcriptions by quantitative PCR (qPCR). **b**, **c** Determination of Wnt5a protein expressions via Western blot analysis. Results are expressed as mean ± SD, *n* = 3. **P < 0.01, ****P < 0.0001

MSCs were transfected with lentiviruses carrying Wnt5a or a control vector, and the overexpression of Wnt5a in MSCs was confirmed at the transcriptional and translational levels. The RT-qPCR analysis confirmed a significantly higher transcription level of Wnt5a in MSCs overexpressing Wnt5a compared to MSCs carrying the control vector (Fig. 3a). Furthermore, Western blot analysis demonstrated upregulated protein expression of Wnt5a in MSCs overexpressing Wnt5a (Fig. 3b and c).

### Overexpression of Wnt5a in MSCs promotes the expression of protective cellular factors for endothelial cell

**Fig. 4:**
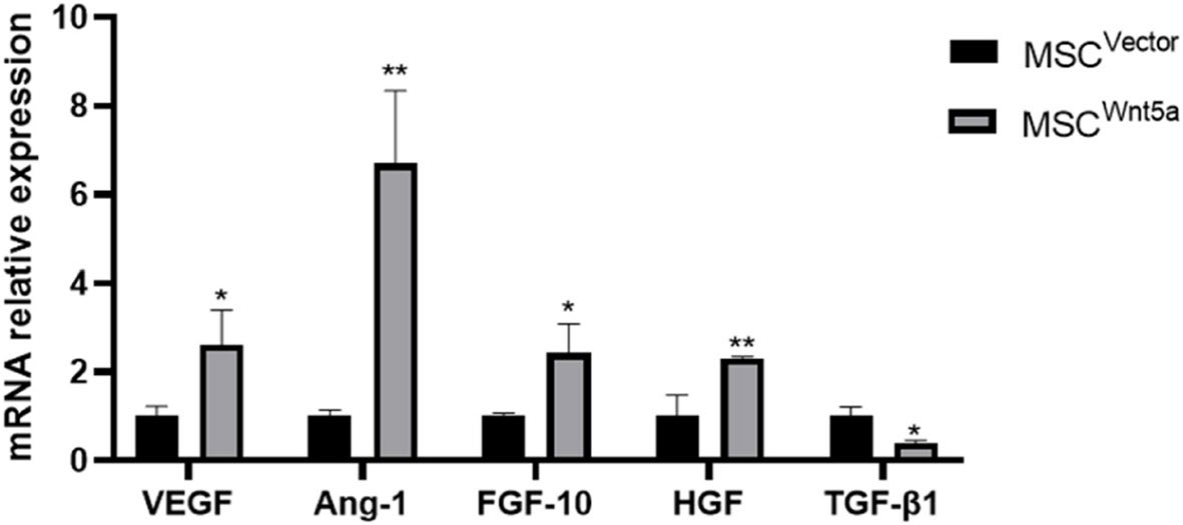
Effects of Wnt5a overexpression on the transcription of protective cytokines in MSCs. The cytokine transcription levels were measured three days after transfection by qPCR analysis. Results are expressed as mean ± SD, *n* = 3. *P < 0.05, **P < 0.01

We analyzed the expression of key cellular factors associated with the protective role of MSC^Wnt5a^. The RT-qPCR array demonstrated that overexpression of Wnt5a upregulated the expression of protective factors, including pro-angiogenic factors VEGF [27] and Ang-1 [28], as well as anti-inflammatory factors FGF-10 [29] and HGF [30]. Additionally, Wnt5a overexpression decreased the expression of the pro-apoptotic cytokine TGF-β1 [31](Fig. 4).

### MSC^Wnt5a^ co-culture promotes endothelial cell migration *in vitro*

**Fig. 5:**
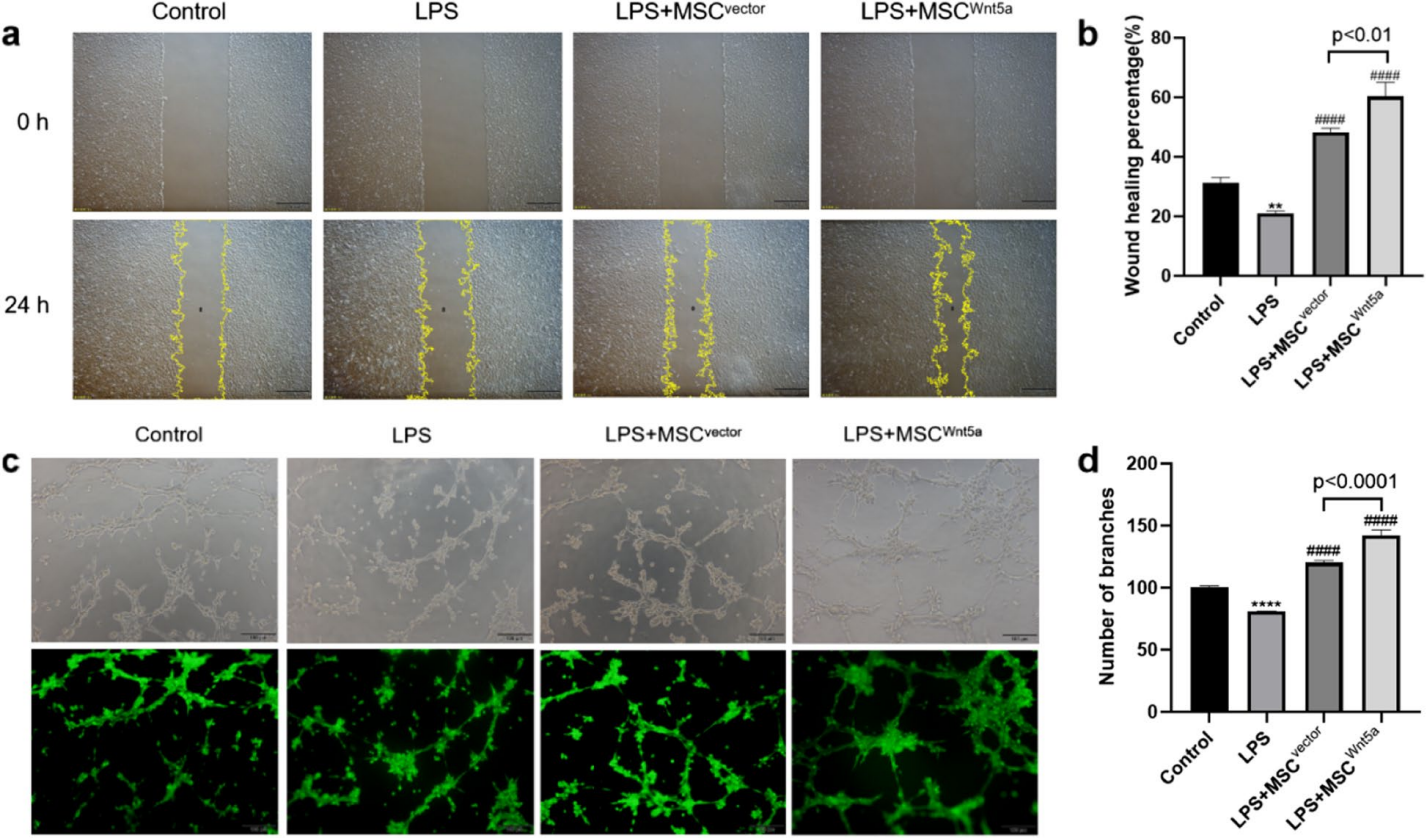
Co-culturing with MSC^Wnt5a^ enhances the migration and angiogenesis of endothelial cells. **a** The representative scratch images of endothelial cells at 0 h and 24 h are presented. **b** Wound healing percentage of endothelial cells with different treatment was shown. **c** The tube formation assay was conducted to detect endothelial cell angiogenesis, and Calcein AM fluorescent dye was used to make the tubular structure more visible. **d** Quantitative analysis found that co-culturing with MSC^Wnt5a^ significantly enhanced endothelial cells angiogenesis compared to the MSC^Vector^ co-culture group and LPS group. Results are expressed as mean ± SD, *n* = 3. **P < 0.01, ****P < 0.0001 compared to control group, ^####^*P* < 0.0001 compared to LPS group

We performed scratch wound healing assay to investigate whether MSC^Wnt5a^ could promote endothelial cell migration. The results demonstrated that LPS stimulation decreased the migration ability of endothelial cells (*P* < 0.01). However, the migration of endothelial cells was significantly increased when co-cultured with different groups of MSCs. Endothelial cells in the MSC^Wnt5a^ co-culture group (*P* < 0.01) migrated farther compared to that in the MSC^Vector^ co-culture group (*P* < 0.01) (Fig. 5a and b). Therefore, MSC^Wnt5a^ was more effective than MSC^Vector^ in promoting in vitro endothelial monolayer wound closure.

### MSC^Wnt5a^ co-culture promotes the tube formation ability of endothelial cells

We next performed a tube formation assay to investigate whether MSC^Wnt5a^ could promote the angiogenesis of endothelial cells. Figure 5c and d showed that the potential for new blood vessel formation by endothelial cells was significantly weaker on the Matrigel in the LPS group compared to the control group (*P* < 0.0001). In contrast, coculturing with either group of MSCs significantly promoted the development of tubular structures. Furthermore, the tube formation ability of endothelial cells was significantly stronger in the MSC^Wnt5a^ co-culture group compared to the MSC^Vector^ co-culture group. The phenotype results were consistent with the upregulation of pro-angiogenic factors in MSC^Wnt5a^.

### MSC^Wnt5a^ Co-culture promotes the proliferation and attenuates the apoptosis of endothelial cells

**Fig. 6:**
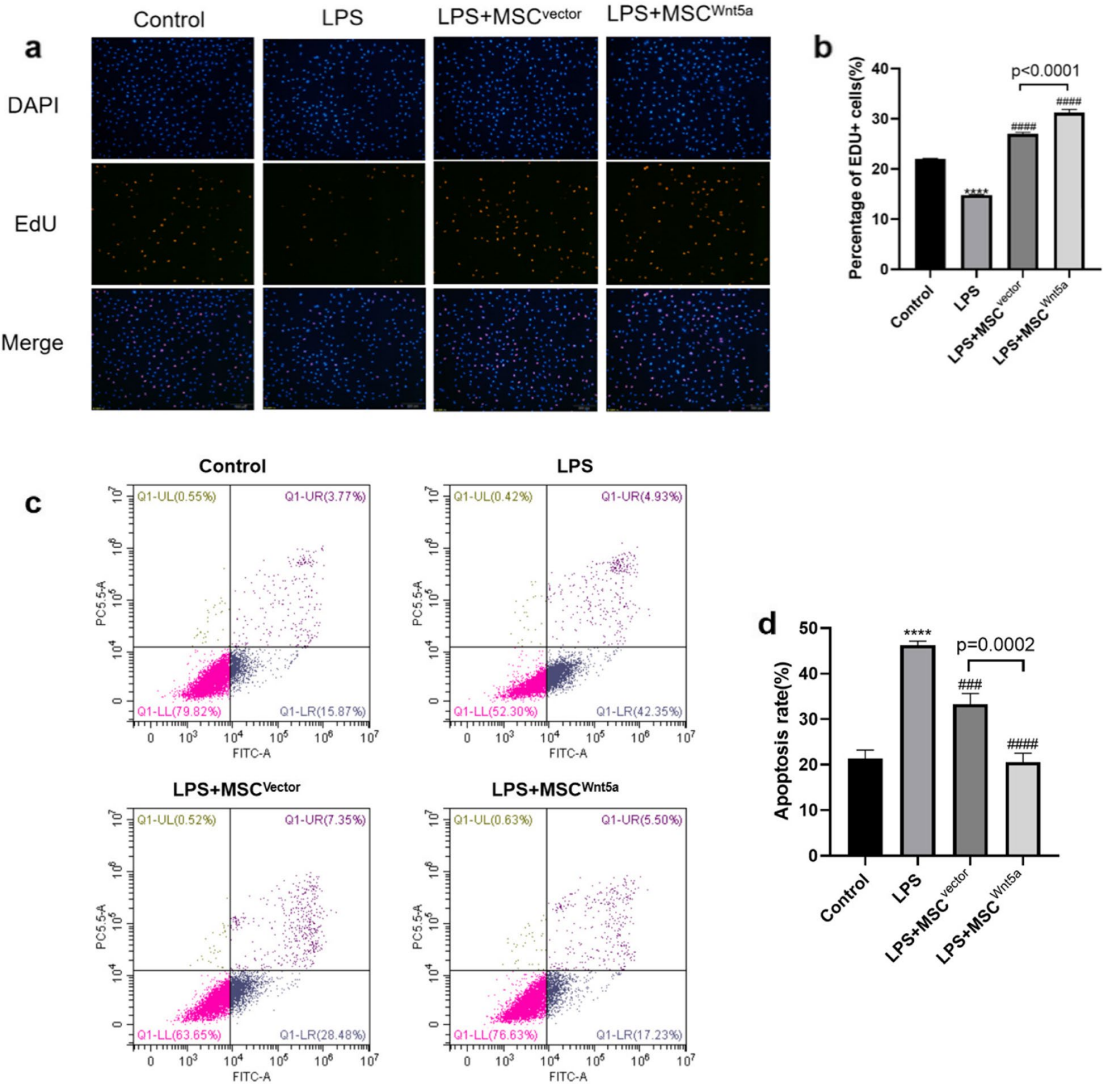
Co-culturing with MSC^Wnt5a^ enhanced proliferation and reduced apoptosis of endothelial cells. **a** The EdU assay was conducted to measure the proliferation of endothelial cells. The blue color labeled nucleus and red labeled actively proliferating cells. **b** Quantitative analysis was performed by counting the ratio of actively proliferating cells. The proportion of proliferation-active cells with various treatment was shown. **c** The Annexin V-FITC/PI assay was performed to detect endothelial cell apoptosis. **d** Quantitative analysis was conducted by summing up the percentages of early apoptotic cells (Q1-LR) and late apoptotic cells (Q1-UR). The proportion of apoptotic cells with various treatment was shown. Results are expressed as mean ± SD, *n* = 3. ****P < 0.0001 compared to control group, ^###^*P* < 0.001, ^####^*P* < 0.0001 compared to LPS group

MSC therapy has proliferative and anti-apoptotic effects. we performed EdU cell proliferation assay and Annexin V-FITC/PI Apoptosis assay to explore the proliferative and anti-apoptotic efficiency of MSC^Wnt5a^ on endothelial cells. The results of the EdU assay (Fig. 6a and b) showed a significant reduction in the proliferation capacity of endothelial cells after LPS treatment compared to the control group (*P* < 0.0001). Similarly, co-culturing with MSCs rescued the proliferation capacity of endothelial cells. Furthermore, MSC^Wnt5a^ co-culturing significantly enhanced the proliferation ability of endothelial cells after LPS-induced injury compared to the MSC^Vector^ co-culture group (*P* < 0.0001). As shown in the apoptosis assay (Fig. 6c and d), LPS stimulation significantly increased the apoptosis rate of endothelial cells compared to the control group (*P* < 0.0001). However, co-culturing with MSC^Vector^ and MSC^Wnt5a^ significantly alleviated the apoptosis level of endothelial cells. Endothelial cells in the MSC^Wnt5a^ co-culture group exhibited a more significant reduction in the apoptosis rate compared to the MSC^Vector^ co-culture group (*P* < 0.01).

### MSC^Wnt5a^ co-culture protects endothelial cells by activating the pi3k/akt signaling pathway

**Fig. 7:**
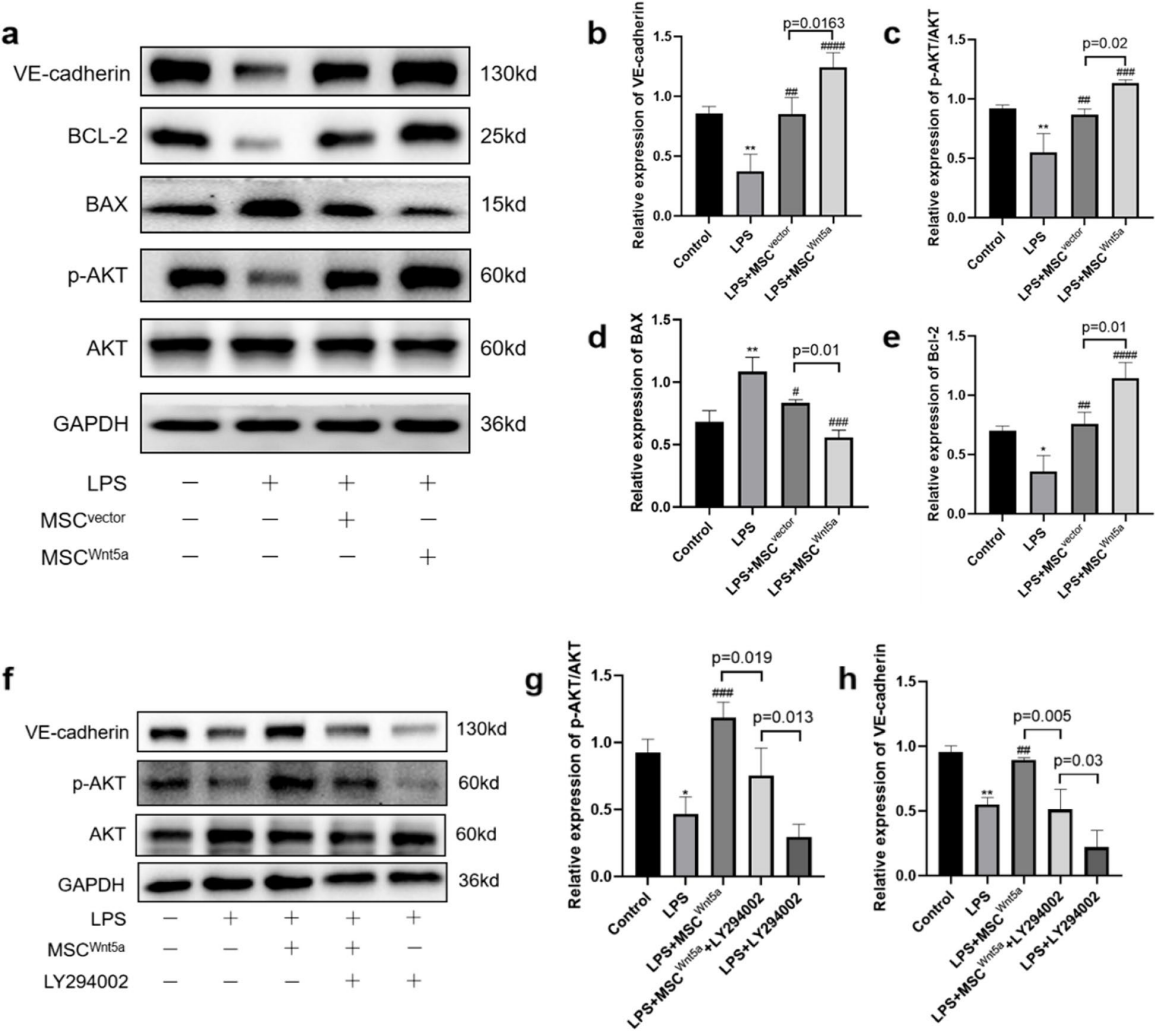
MSC^Wnt5a^ co-culture activates the PI3K/AKT signaling in endothelial cells. **a** The expression of AKT/p-AKT, VE-cadherin and apoptosis-related proteins (BCL-2 and BAX) were evaluated by Western blotting. **b**-**e** Densitometric analysis of Western blots. GAPDH served as an internal reference. **f **Detecting the effect of LY294002 on MSC^Wnt5a^ co-culture induced changes in the PI3K/AKT pathway via Western blotting. **g**-**h** Densitometric analysis of Western blots. GAPDH served as an internal reference. Results are expressed as mean ± SD, *n* = 3. *P < 0.05, **P < 0.01, ***P < 0.001 compared to control group. ^#^*P* < 0.05, ^##^*P* < 0.01, ^###^*P* < 0.001, ^####^*P* < 0.0001 compared to LPS group

It was reported that Wnt5a promoted the migration and proliferation of cancer cells by activating the PI3K/AKT signaling [32–34]. Therefore, we tried to determine if MSC^Wnt5a^ protected endothelial cells through the PI3K/AKT signaling pathway. In order to investigate the protective mechanism of MSC^Wnt5a^ on endothelial cells, we performed western blot assay to determine the expression of phosphorylated AKT, VE-cadherin, Bcl-2 and BAX (Fig. 7a). AKT protein phosphorylation was significantly reduced in the LPS group compared to the control group, suggesting that LPS-induced endothelial cell injury is linked to PI3K/AKT signaling inhibition. Furthermore, VE-cadherin and BCL-2 expression were significantly decreased in the LPS group, whereas the expression of BAX was up-regulated. In comparison to the LPS group, the expression of phosphorylated AKT, VE-cadherin and BCL-2 in endothelial cells were higher in both the MSC^Vector^ co-culture group and the MSC^Wnt5a^ co-culture group, while the expression of BAX was decreased. Moreover, the changes in these proteins were more pronounced in the MSC^Wnt5a^ co-culture group compared to the MSC^Vector^ co-culture group, suggesting that MSC^Wnt5a^ had stronger protective effects on endothelial cells than MSC^Vector^ (Fig. 7b-e). Next, we used LY294002 to block the PI3K/AKT signaling in endothelial cells. The effects of MSC^Wnt5a^ co-culture on AKT protein phosphorylation and VE-cadherin expression were attenuated (Fig. 7f-h). These results indicated that MSC^Wnt5a^ co-culture activates the PI3K/AKT signaling in endothelial cells.

**Fig. 8:**
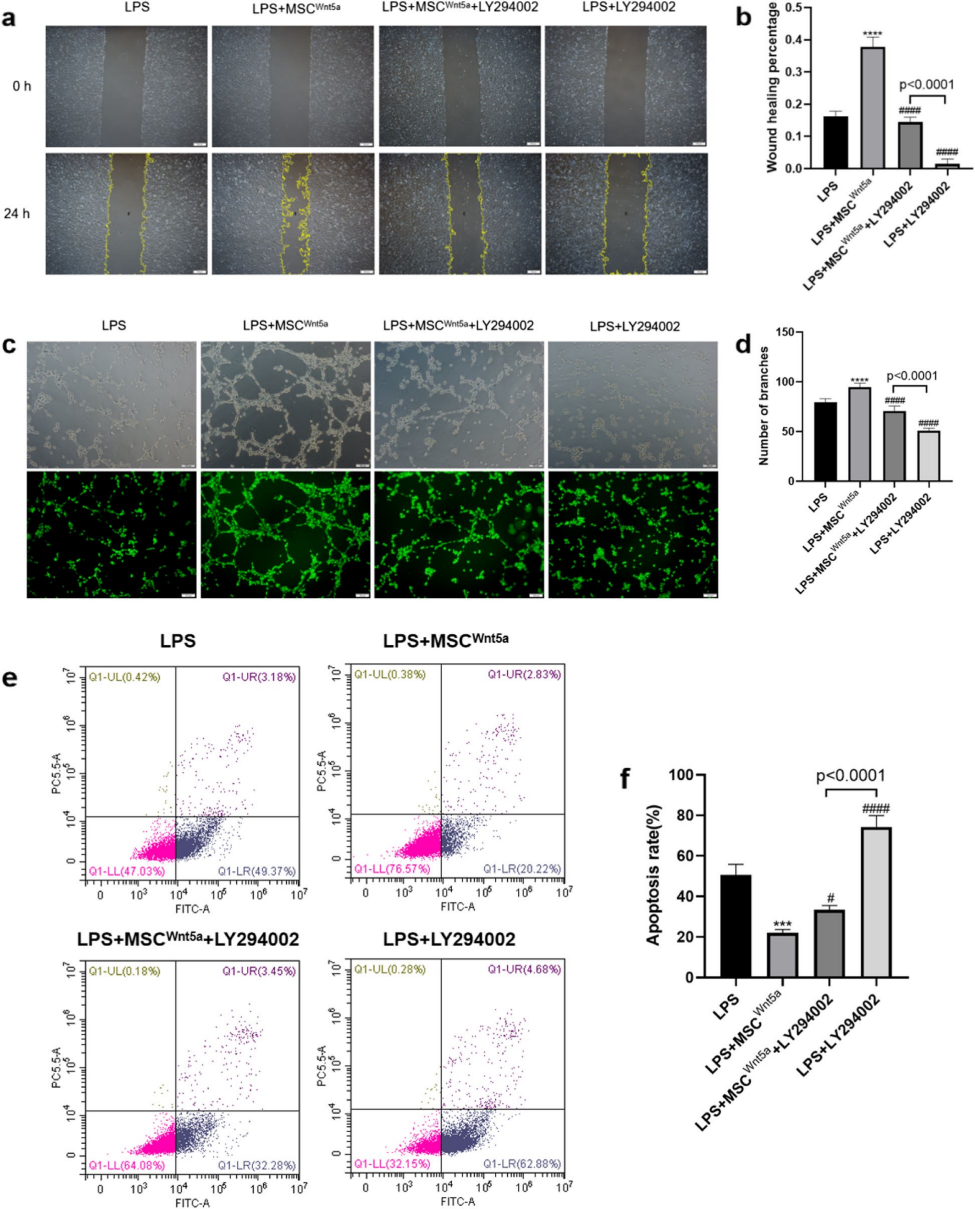
LY294002 abolished the protective effects of MSC^Wnt5a^ on endothelial cells. **a** The representative scratch images of endothelial cells at 0 h and 24 h are presented. **b** Wound healing percentage of endothelial cells with different treatment was shown. **c** The tube formation assay was conducted to detect endothelial cell angiogenesis, and Calcein AM fluorescent dye was used to make the tubular structure more visible. **d** Quantitative analysis of vascular brunching number with different treatment was counted. **e** Annexin V-FITC/PI was used to detect endothelial cell apoptosis. **f** Quantitative analysis was conducted by summing up the percentages of early apoptotic cells (Q1-LR) and late apoptotic cells (Q1-UR). The proportion of apoptotic cells with various treatment was shown. Results are expressed as mean ± SD, *n* = 3. ****P < 0.0001 compared to LPS group. ^####^*P* < 0.0001 compared to MSC^Wnt5a^ group

Further, we assessed if LY294002 blocked the protective effects of MSC^Wnt5a^ on endothelial cells. We stimulated endothelial cells with LPS and LY294002, the pro-migration and angiogenic effects of MSC^Wnt5a^ were attenuated by LY294002 (Fig. 8a-d). Meanwhile, LY294002 treatment also partially reversed the anti-apoptotic effect of MSC^Wnt5a^ co-culture on endothelial cells (Fig. 8e-f). In summary, the above results demonstrate that Wnt5a overexpression optimized the protective effects of MSCs against LPS-induced endothelial cell injury by activating the PI3K/AKT signaling pathway.

## Discussion

Microvascular endothelial cells are crucial in maintaining pulmonary microcirculation. Accumulating researches have demonstrated the significant protective effects of MSCs for endothelial injury. Microvascular endothelial cell-derived Wnt5a is essential for angiogenesis, and its absence inhibits vascular structure formation [22]. We discovered that overexpression of Wnt5a promoted the therapeutic value of MSCs for endothelial injury through activation of the PI3K/AKT signaling. Therefore, it was considered a promising strategy to overexpress Wnt5a in MSCs for the treatment of endothelial injury.

In recent years, researchers have extensively studied the crucial role of Wnt5a in endothelial cell function. For instance, Wnt5a secreted by monocytes can bind and activate its receptor FZD5 in microvascular endothelial cells and induce angiogenesis [35]. Similarly, interleukin-1β induces upregulation of Wnt5a in endothelial cells, leading to enhanced cell migration [36]. Additionally, Wnt5a increased endothelial cell proliferative activity and upregulated the expression of angiogenic genes MMP-1 and Tie-2 [23]. However, some studies have suggested contrary findings. For example, Wnt5a silencing significantly suppresses IL-4-induced endothelial barrier dysfunction [37], and the Wnt5a antagonist prevents the disruption of VE-cadherin adherent junctions and protects the barrier function in cardiac endothelium [38]. The paradoxes may arise from variations in cell types, stimulus conditions, and experimental approaches. Nevertheless, the aforementioned findings suggested the crucial role of Wnt5a in endothelial function. Studies have reported the protective effects of MSC-based therapies, particularly in alleviating vascular endothelial injury in sepsis and ALI [8, 39]. Furthermore, combining gene therapy and MSC therapy would further enhance the protective effects of MSCs on ALI/ARDS [40]. Moreover, activation of the Wnt5a/FZD5 signaling pathway promotes MSC proliferation, potentially improving the efficacy of MSC-based therapies [41]. In our study, MSC^Wnt5a^ demonstrated stronger promotion of proliferation, migration, angiogenesis, and inhibition of apoptosis in endothelial cells compared to MSC^Vector^. This further confirmed the protective value of Wnt5a in endothelial function. As a result, the findings suggested that Wnt5a overexpression could enhance the protective efficacy of MSCs against endothelial injury.

In the present study, we discovered that MSC^Wnt5a^ activates the PI3K/AKT signaling, and develops anti-apoptotic, proliferative, and tubulogenic effects in LPS-stimulated endothelial cells. Our previous study demonstrated that ghrelin attenuates LPS-induced endothelial barrier disruption through the PI3K/AKT pathway [42]. PI3K/AKT activation also increases NO production, promotes the function of endothelial cells [43, 44]. We observed that MSC^Wnt5a^ co-cultured activates PI3K/AKT signaling in endothelial cells, promotes expression of Bcl-2 and inhibits BAX expression. The percentage of EdU-positive endothelial cells increased, while the rate of apoptotic cells decreased. Disruption of VE-cadherin adherent junctions increases endothelial permeability, with protein and inflammatory cells seeping out from small vessels [45]. Additionally, we observed upregulation of VE-Cadherin in endothelial cells co-cultured with MSC^Wnt5a^, suggesting a potential protective role in maintaining the endothelial barrier. Furthermore, we treated endothelial cells with the PI3K/AKT inhibitor LY294002 and observed that the effects of MSC^Wnt5a^ on AKT phosphorylation and VE-Cadherin upregulation were abolished. This further validates the activation of PI3K/AKT signaling in MSC^Wnt5a^ therapy for endothelial injury.

MSC therapy has been proven as a prospective method for treating ALI/ARDS. However, reduced cell activity in an inflammatory environment and limited therapeutic effect are the main obstacles for this therapy. In this study, we genetically overexpressed Wnt5a in MSCs for the first time and demonstrated that MSC^Wnt5a^ enhances the therapeutic potential of MSCs on endothelial injury, thereby optimizing the use of MSCs in ALI/ARDS. However, the limitations of our research cannot be ignored. First, we used LPS to stimulate an endothelial cell line in vitro, but this may not fully mimic the effects of various pathogens (bacteria, viruses, fungi, or mycobacteria) that can damage the vascular endothelium and the pathophysiological environment to which endothelial cells are exposed [46, 47]. Second, Wnt5a overexpression may alter the secretion profile of other paracrine substances, as evidenced by changes in the transcription of cellular factors in MSC^Wnt5a^. Further RNA sequencing is needed to investigate potential paracrine factors, other than Wnt5a, that contribute to the therapeutic effects of MSC^Wnt5a^. Third, it is unclear whether Wnt5a activates the PI3K/AKT pathway directly or indirectly, which requires further exploration in future studies.

## Conclusions

In summary, our study demonstrated that MSC^Wnt5a ^co-culture promotes the proliferation, migration, angiogenesis and inhibits apoptosis of endothelial cells subjected to LPS stimulation. And MSC^Wnt5a ^activates the PI3K/AKT pathway in endothelial cells to develop the protective effects. Consequently, our findings have provided a novel theoretical foundation for the treatment of ALI/ARDS. And we need to run clinical trials to further investigate whether MSC^Wnt5a^-based cell therapy is a promising treatment strategy for ALI/ARDS.

### Supplementary Information

**Supplementary Material 1.**

## Data Availability

The raw data used to support the findings of this study can be acquired by contacting the corresponding author, Mian Zeng.

## Abbreviations

ALI: Acute lung injury
ARDS: Acute respiratory distress syndrome
MSCs: Mesenchymal stem cells
LPS: Lipopolysaccharide
HOXB4: Homeobox B4
MEM-α: Minimum Essential Medium Alpha
DMEM: Dulbecco's modified Eagle's medium
FBS: Fetal bovine serum
PBA: PBS containing 1% bovine serum albumin
EdU: 5-Ethynyl-20-deoxyuridine
qPCR: Quantitative Real-time PCR
GAPDH: Glyceraldehyde-3-phosphate dehydrogenase
Wnt5a: Wingless-related integration site family member 5a
VEGF: Vascular endothelial-derived growth factor
Ang-1: Angiotensin-1
FGF-10: Fibroblast growth factor-10
HGF: Hepatocyte growth factor
TGF-β1: Transforming growth factor-beta1
