# Supplementary material for: Overexpression of Wnt5a promoted the protective effect of mesenchymal stem cells on Lipopolysaccharide-induced endothelial cell injury via activating PI3K/AKT signaling pathway
Source: BMC Infect Dis. 2024 Mar 20;24:335. doi: 10.1186/s12879-024-09204-4 (PMC10953236; doi:10.1186/s12879-024-09204-4)
Supplement: Supplementary file 1 — Supplementary Material 1. [file 12879_2024_9204_MOESM1_ESM.pdf]

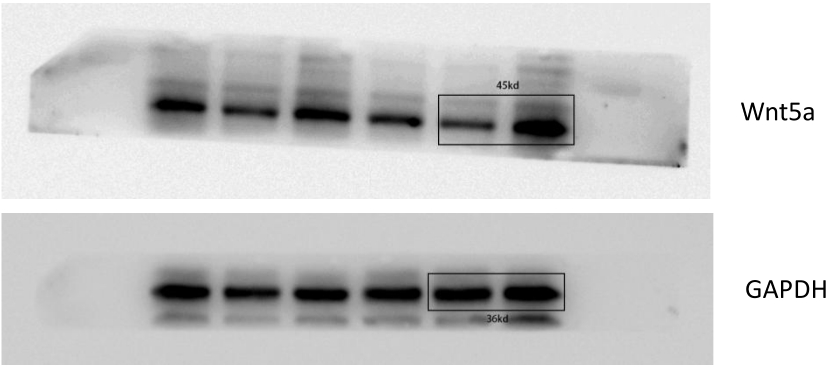

Fig. S1: Validation of MSC^{Wnt5a}.

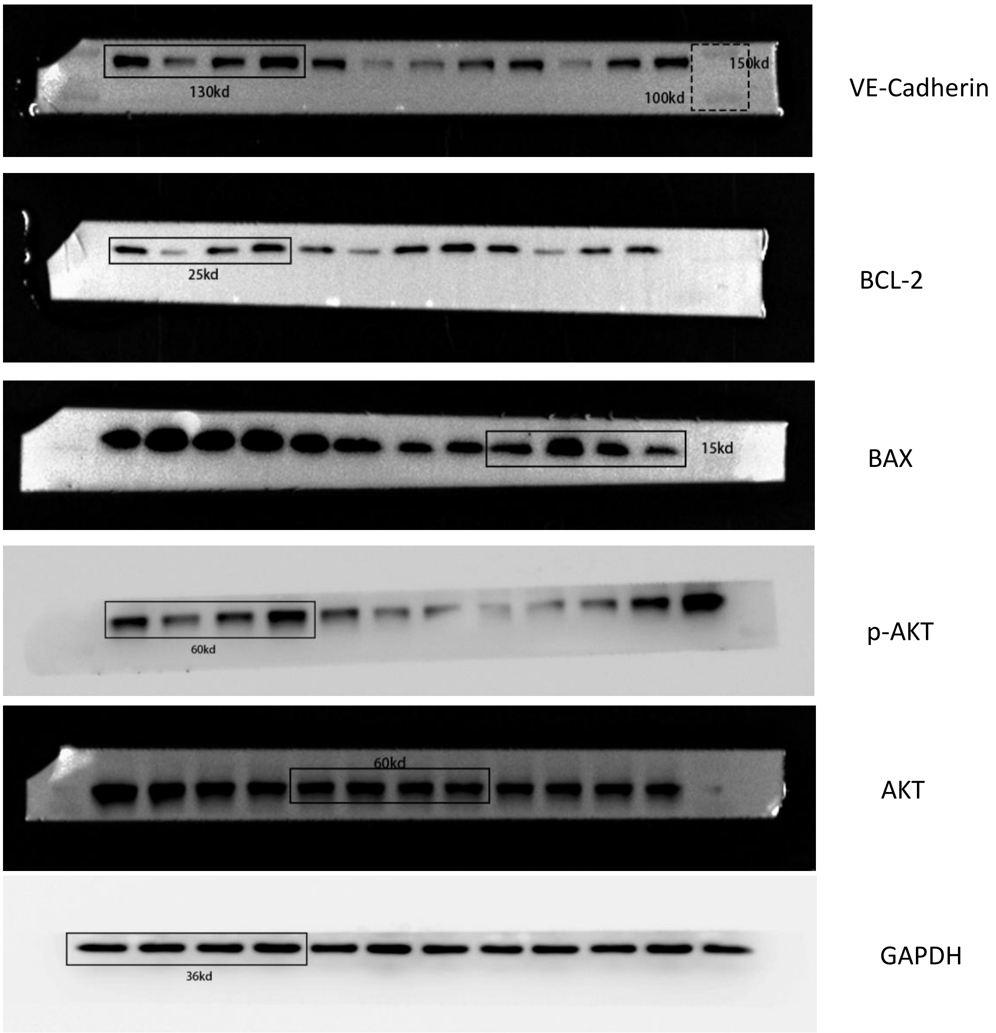

Fig. S2: MSC^{Wnt5a} co-culture activates the PI3K/AKT signaling in endothelial cells.

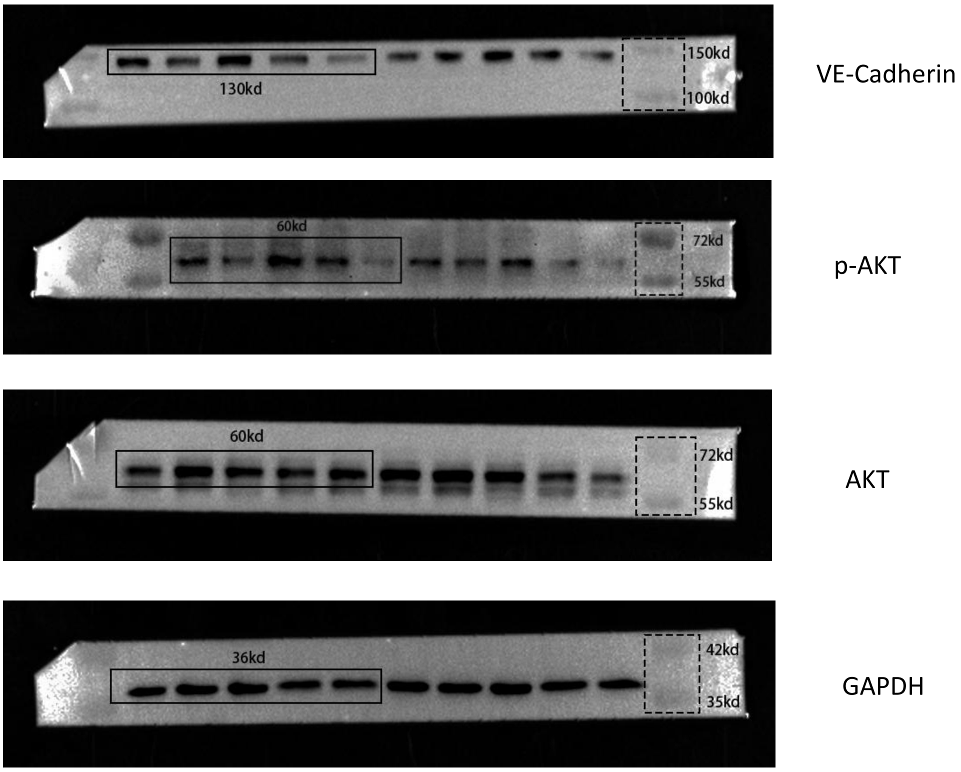

Figure. S3: MSC^{Wnt5a} co-culture activates the PI3K/AKT signaling in endothelial cells.
